# A Delphi survey-based development and validation of cognitive-communication assessment tool for Indian preschoolers

**DOI:** 10.1590/2317-1782/20232022309

**Published:** 2023-10-27

**Authors:** Aparna Prasanna, Gagan Bajaj, Malavika Anakkathil Anil, Jayashree S. Bhat

**Affiliations:** 1 Department of Audiology and Speech-Language Pathology, Kasturba Medical College, Mangalore, Manipal Academy of Higher Education, Manipal, Karnataka, India.; 2 The MARCS Institute of Brain, Behaviour and Development, Western Sydney University - Westmead, Australia.; 3 Nitte Institute of Speech and Hearing - Mangalore, Karnataka, India.

**Keywords:** Child Development, Short Term Memory, Executive Functions, Cognitive Tests, Preschool Children

## Abstract

**Purpose:**

To address the need for a standardized assessment tool for assessing cognitive-communication abilities among Indian preschoolers, the current study aimed at describing a Delphi based development and validation process for developing one such tool. The objectives of the research were to conceptualize and construct the tool, validate its content, and assess its feasibility through pilot testing.

**Methods:**

The study followed a Delphi approach to develop and validate the tool across four phases i.e. conceptualization; construction; content validation; and pilot testing. The first three phases were performed with a panel of six experts including speech-language pathologists and preschool teachers while the pilot testing was done with 20 typically developing preschoolers. A literature review was also conducted with the Delphi rounds to support the developmental process.

**Results:**

The first two rounds of the Delphi aided in the construction of a culturally and linguistically suitable story-based cognitive-communication assessment tool with the memory (free recall, recognition, and literary recall) and executive function (reasoning, inhibition, and switching) related tasks relevant for preschoolers. The content validation of the tool was continued with the experts till the revisions were satisfactory and yielded an optimum Content Validity Index. The pilot test of the finalized version confirmed its feasibility and appropriateness to assess developmental changes in the cognitive-communication abilities of preschoolers.

**Conclusion:**

The study describes the Delphi-based conceptualization, construction, content validation, and feasibility check of a tool to assess cognitive-communication skills in preschool children.

## INTRODUCTION

Cognitive and communicative processes interact to enable individuals to understand the world. Such an interaction between cognition and communication, termed cognitive-communication, involves mental processes, such as attention, memory, problem-solving, reasoning, and other executive functions that mediate communication^(1)^. The development of these cognitive-communication skills is critical for an individual's daily life success, and its development begins as early as the preschool period.

The preschool period witnesses the most significant annualized changes in the brain's anatomical and physiological characteristics^(2)^, resulting in rapid developmental changes across the cognitive-communication skills^(3,4)^. Developing cognitive-communication skills during preschool is crucial since it forms the foundation for other critical domains, such as language and literacy^(5)^. Research shows that the cognitive-communication skills of a child, such as memory and executive functions, facilitate the learning process and significantly contribute to academic success^(4)^. Given the high impact of these skills on learning and life success, assessing cognitive-communication competence and providing intervention, if needed, to prevent negative consequences as early as preschool age is vital.

In preschoolers, the cognitive-communication skills are usually assessed either as a part of standardized IQ tests or as specific tasks for each cognitive-communication skill^(6)^. However, the existing tools and tasks being normed and standardized in high-income countries, such as the western population, limit their applicability to eastern countries, such as India. India is a lower-middle-income country with a unique feature of diversities across religious, social, and cultural backgrounds setting a prime example of 'unity in diversity.’ This diverse background shapes India's educational system and child development in the Indian context^(7)^. Therefore, cognitive development which is influenced by such contextual differences cannot be fully assessed and understood unless the cultural context is considered during the assessment^(8)^. Western normed tests impose several challenges, such as bias in assessment caused by unfamiliar vocabulary, stimulus/materials, test requirements, situations, methods of assessment, and cultural variations when used in the non-Western context^(9)^. It is vital that preschoolers’ assessments be culturally, contextually, and linguistically sensitive^(10)^. The materials and assessment methods used for preschoolers need to reflect the cultural or national framework to better represent their capabilities^(10)^. Moreover, the existing tools in preschoolers predominantly target cognitive skills, thereby missing to reflect the cognitive-communication skills used in a real-world everyday communication context, and the literature report that an assessment tool of cognitive-communication skills needs to include the characteristics of a communication context.

Due to the scarcity of such standardized tools in India that assess cognitive-communication skills in preschoolers tailored to the cultural and linguistic contexts, and recalling the importance of identifying children with difficulties during preschool when the brain is more plastic to prevent negative consequences in a child's life, the current study was undertaken. The present study aimed at describing a Delphi survey-based development and validation of a cognitive-communication assessment tool for Indian preschoolers. The specific objectives of the research were to conceptualize and construct the tool, validate its contents, and examine its feasibility through pilot testing.

## METHODS

**Figure 1:**
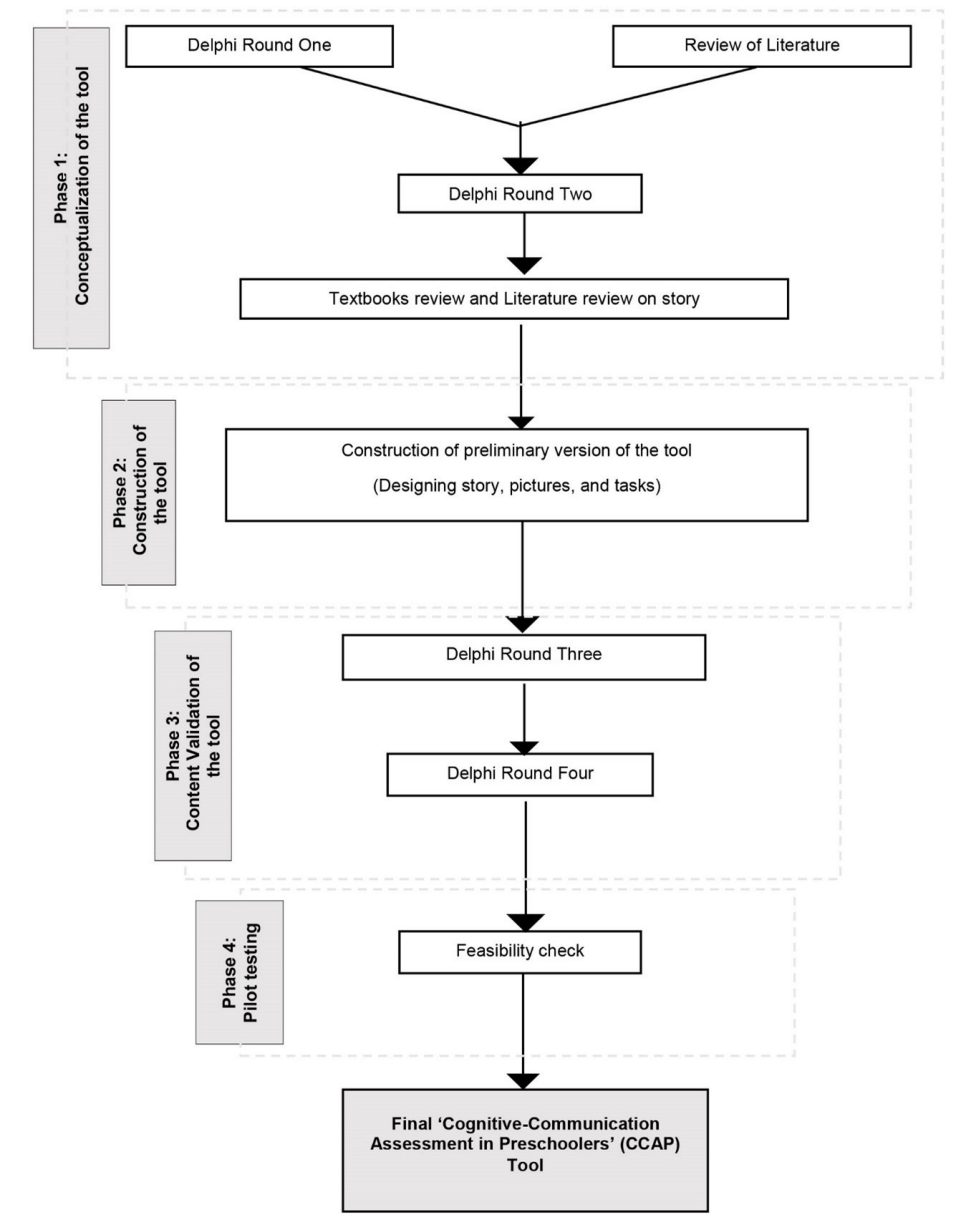
Phases of Tool Development

The study was initiated after approval from the Institutional Ethical Committee (IEC KMC MLR 02-2020/62). This study used the Delphi method, an iterative process of collecting anonymous judgments of experts using a series of questions and analysis techniques interspersed with controlled feedback^(11)^ for tool development. The reliability of the method was verified using the checklist proposed by Hasson et al.^(12)^. The study was conducted in four phases: the first phase focused on tool conceptualization, the second phase involved tool construction, and the third phase was dedicated to content validation, followed by pilot testing during the fourth phase. The phases of tool development are illustrated in Figure 1.

### Phase one: Conceptualization of the tool

This phase aimed to conceptualize the tool by identifying the critical cognitive-communication domains and stimuli/tasks that could be included in the tool to assess the preschooler's cognitive-communication skills. The conceptualization process was accomplished through two rounds of Delphi and literature review. A panel of six subject experts, consisting of three Speech-Language Pathologists (SLPs) and three preschool teachers, were selected as participants using purposive sampling. It was ensured that the SLPs held a post-graduate degree or Ph.D. in Speech-Language Pathology and were actively involved in cognitive-communication research with a minimum of five years of clinical experience in the field of cognitive-communication among children. For the preschool teachers, it was ascertained that they had a minimum of five years of preschool working experience with adequate knowledge of the cognitive-communication skills among preschoolers. The participants included were from wide range in years of experience from five to more than twenty years with a distribution of one expert with five to ten years, three with ten to twenty years, and two with more than twenty years of experience. Two of the subject experts were males and four were females. Detailed demographics of the experts are presented in Table 1. Written consent was obtained from all experts after informing them of the purpose of the study, the estimated time required, the participation criteria, their need to stay engaged in the research process, and the voluntary nature of the Delphi study. A unique code was assigned to the agreed participants to ensure the confidentiality and anonymity of the expert panel. Each expert was then asked for their preferred modality in the Delphi round questionnaires (printed or mail version) and was contacted accordingly.

**Table 1:** Delphi Expert Panel Details

Characteristics	Number of Participants
**Profession**	3 SLPs
	3 Preschool teachers
**Gender**	2 Males (1 SLP,1 Preschool teacher)
	4 Females (2 SLPs, 2 Preschool teachers)
**Years of working experience**	
5-10 years	1
10-20 years	3
>20 years	2
**Qualification**	
Master's degree in Speech-Language Pathology	1
Doctoral degree in Speech-Language Pathology	2
Bachelor's degree in education with specialized training teaching preschoolers	3

#### Delphi round one

The first Delphi round focused on collecting experts' opinions regarding the necessity of developing a tool, the critical cognitive-communication domains to be assessed, and stimuli or materials that need to be used for assessment in the tool among preschool children. A questionnaire containing six open questions was prepared for this purpose and shared with the experts (Table 2). The experts were given a two-week period, with a reminder in between to complete the questionnaire. The responses obtained from the experts were qualitatively analyzed by the researchers, and a summary of the results was provided to the participants as controlled feedback of the Delphi procedure to inform the participants on other participants’ perspectives and provide an opportunity to clarify or change their views.

**Table 2:** Contents of Delphi Round One Questionnaire

**Q .No.**	**Question**
1	Describe your views regarding the necessity for assessing cognitive-communication skills in preschoolers
2	Explain various methods /stimuli which could be used to assess cognitive-communication skills in preschoolers
3	Describe your views about the applicability of such tools in the Indian context. How the existing tools are linguistically and culturally appropriate for the Indian context
4	Elaborate on critical cognitive-communication domains that could be assessed among preschoolers
5	Provide your views regarding the nature of stimuli/material which could be used to assess the preschooler's cognitive-communication skills

#### Literature review on cognitive-communication in preschoolers

An extensive review of literature on cognitive-communication among preschoolers was also conducted to conceptualize the tool^(13,14)^. The review provided insight into the importance of assessing cognitive-communication skills in preschoolers, the critical cognitive-communication skills that need to be assessed in preschoolers, and the range of critical stimuli/tasks that could be used to assess the cognitive-communication skills among preschoolers.

Based on the findings during the first Delphi round and the literature review, the necessity for assessing cognitive-communication among preschoolers was confirmed wherein 'Memory’ and ‘Executive Function' were identified as the salient assessment domains, and 'stories' emerged as the consensual stimuli that could adequately assess cognitive-communication skills during the preschool years.

#### Delphi round two

The second round of the Delphi was conducted to obtain deeper insights into the suggested stimuli and domains for the present tool. The second round of the Delphi contained four open-ended questions eliciting experts' perspectives about 'Stories' as the potential stimuli and 'Memory and Executive Function' as central domains of assessment (Table 3). Similar to the first round of Delphi, the experts were provided with one reminder at the end of the first week to complete and return the questionnaires to the researchers. The responses were analyzed qualitatively and discussed among the researchers, and controlled feedback on the outcome was provided.

**Table 3:** Contents of Delphi Round Two Questionnaire

**Q .No.**	**Question**
1	Describe your views on the nature of stories that could be used to assess cognitive communication in preschoolers
2	Describe your views on various aspects to be controlled while constructing stories to assess cognitive communication in preschoolers.
	· What should be the length of the story?
	· What modality is to be used for story presentation?
	· What kind of vocabulary can be used in the story?
3	Provide suggestions on the nature of tasks that could be used with stories assessing memory in preschoolers.
4	Provide suggestions on the nature of tasks that could be used with stories assessing executive function in preschoolers.

#### Literature review on story construction and preschool textbook reviews

Following the advice of experts during the second round of Delphi, a literature review on story construction along with a review of preschool textbooks was conducted as the final step in conceptualizing the tool. The literature review provided additional insights into the research evidence pertaining to several factors to be considered when constructing a story for preschoolers^(15-17)^. The review of preschool textbooks helped to extract age-appropriate vocabulary and inputs regarding the kinds of stories and activities relevant to preschool children.

Based on the findings obtained from the reviews and Delphi rounds, a story-based tool was conceptualized that needed to be developed while considering a novel, real-life, Indian context-based theme with assessment tasks tapping the memory (free recall, recognition, and literary recall) and executive function domains (reasoning, inhibition, and switching).

### Phase two: Construction of the tool

The second phase aimed at constructing a novel story based on real-life context-based sequences that could be understood and explained by a causal chain of events in the mind. Six pictures corresponding to the story content were designed and drawn by an artist. Three tasks assessing memory and two assessing executive function were designed based on the story to assess cognitive-communication skills. The memory tasks included a free recall task that required children to recall the story in any order, a recognition task that required children to identify the words/pictures of the story from a list of four words/picture choices, and a literary recall task that required children to answer Wh-questions based on the story. The executive function tasks included a reasoning task, which required children to answer open-ended questions that tap reasoning abilities across explanations^(18)^, prediction^(19)^, inference^(20,21)^, and inhibition and switching task, designed similar to the task used by Espy^(22)^, which required children to name, inhibit, switch, and inhibit and switch together under four conditions of picture presentations. For all constructed tasks, the correct responses were scored as one, and incorrect responses were scored as zero. The tool was constructed in English, considering the medium of instruction in the majority of Indian schools. Attention was given to equally distributing the story elements across the tasks and modality of presentation of the story.

The initial draft of the story-based tool was discussed among the investigators and underwent a series of modifications. The modified story was then recorded as a narrative in three sections, with two pictures depicting each of the three sections.

### Phase three: Content validation of the tool

This phase aimed to validate the preliminary version of the story-based tool through another round of Delphi. An expert panel of six members with similar inclusion criteria as the participants in the first and second Delphi rounds was formed to validate the content of the developed tool.

#### Delphi round three

In the third round of Delphi, a questionnaire was prepared for content validation of the preliminary version of the tool (story, pictures, and tasks) and distributed among experts along with the developed tool. The experts were requested to complete and return the questionnaire to the researcher. The questionnaire contained closed-ended questions regarding content relevance, linguistic appropriateness, demand for the child, instruction and scoring appropriateness, and comprehensibility of the content, pictures, and tasks. The experts were asked to rate the contents on a 5-point Likert scale where one stands for Highly Inappropriate / Highly incomprehensible /Highly irrelevant, two for “Inappropriate /Incomprehensive /Irrelevant,” three for “Not sure,” four for Appropriate /Comprehensive /Relevant, and five for “Highly Appropriate /Highly comprehensive /Highly relevant.” The questionnaire also had a provision for any further suggestions regarding the content. The experts were also provided additional written information about the summary of the phases, which led to the generation of a preliminary version of the tool. Appropriate instructions were given for the rating, and two weeks were given to respond with one reminder at the end of the first week.

#### Delphi round four

The investigators quantitatively and qualitatively analyzed the results obtained during the third round of Delphi. Consensus on the agreement was calculated, and the prior measure of agreement was set at 80%. Contents that obtained greater than 80% consensus on the agreement were maintained, and those that obtained less than 20% were excluded. Contents that reached a consensus on the agreement between 80% and 20% were revised based on ratings and comments. The revised version of the tool was then redistributed to the participants during the fourth round of the Delphi. The questionnaire used during the fourth round of the Delphi included only a content evaluation of the revised contents, and experts were requested to rate the contents similarly to the third round. The responses obtained during this round were analyzed quantitatively and qualitatively, and the results were summarized to experts for controlled feedback. Based on the finalized ratings provided by the experts, the content validity index was calculated at item and scale level^(23)^. The Item level Content Validity Index (I-CVI) was calculated by dividing the number of experts who rated the items as either four or five by the total number of experts and the Scale level Content Validity Index (S-CVI) was computed by taking the average of I-CVI across each task. The values obtained were interpreted using the Lynns criteria for content validation which considered the standard error and recommended a minimum of 0.78 I-CVI and S-CVI of 0.90 or higher to have excellent content validity when rated by six to ten subject experts^(23)^. Correspondingly, items with a content validity index of greater than 0.78^(23)^ were retained in the final tool.

The third and fourth rounds of the Delphi aided in achieving consensus among expert panelists regarding the adequate face and content validity of the tool. Thus, the Delphi procedure was concluded in the fourth round, and the content-validated tool was presented and shared among the panelists with the acknowledgement for their contributions.

### Phase four: Pilot testing

To check the feasibility of the developed tool, a pilot study was conducted by administering it to twenty typically developing preschool children between 3.6 years and 5.5 years^(24)^ attending English medium schools in the Dakshina Kannada district of Karnataka. The participants were equally divided into two groups based on their age [Group I:3.6-4.5 years (Mean age = 3.8 years, Standard Deviation = 0.26) and Group II:4.6-5.5 years (Mean age = 5.1 years, Standard Deviation = 0.19)] with each group having similar gender representation. The participants were recruited based on the selection criteria similar to Prasanna et al.^(25)^ confirming the typical development (Ten Question Screen and Assessment of Language Development) and middle socio economic status based on the parental occupation and education (Modified Kuppuswamy Socio-economic Scale). Informed written consent was obtained from the school authorities and parents of the participants before the tool was administered. The participants were seated in a quiet room at home, and the recorded story was narrated in three sections using a laptop and headphones. Each section of the story was followed by an assessment of memory and executive functions. The entire assessment took approximately one hour per participant. The results obtained for these tasks were compiled and subjected to inferential statistics using Independent t-test or Mann-Whitney U test based on the normality of the data in the respective tasks to verify the feasibility of the tool. The tool was considered to be feasible only if all the children included in the pilot study could comprehend the instructions and complete the tasks within one hour without any disruptions or fatigue. Furthermore, it was important that the tool must be able to track the changes in cognitive-communication skills across the age range in the pilot study for it to be considered feasible.

## RESULTS

**Figure 2:**
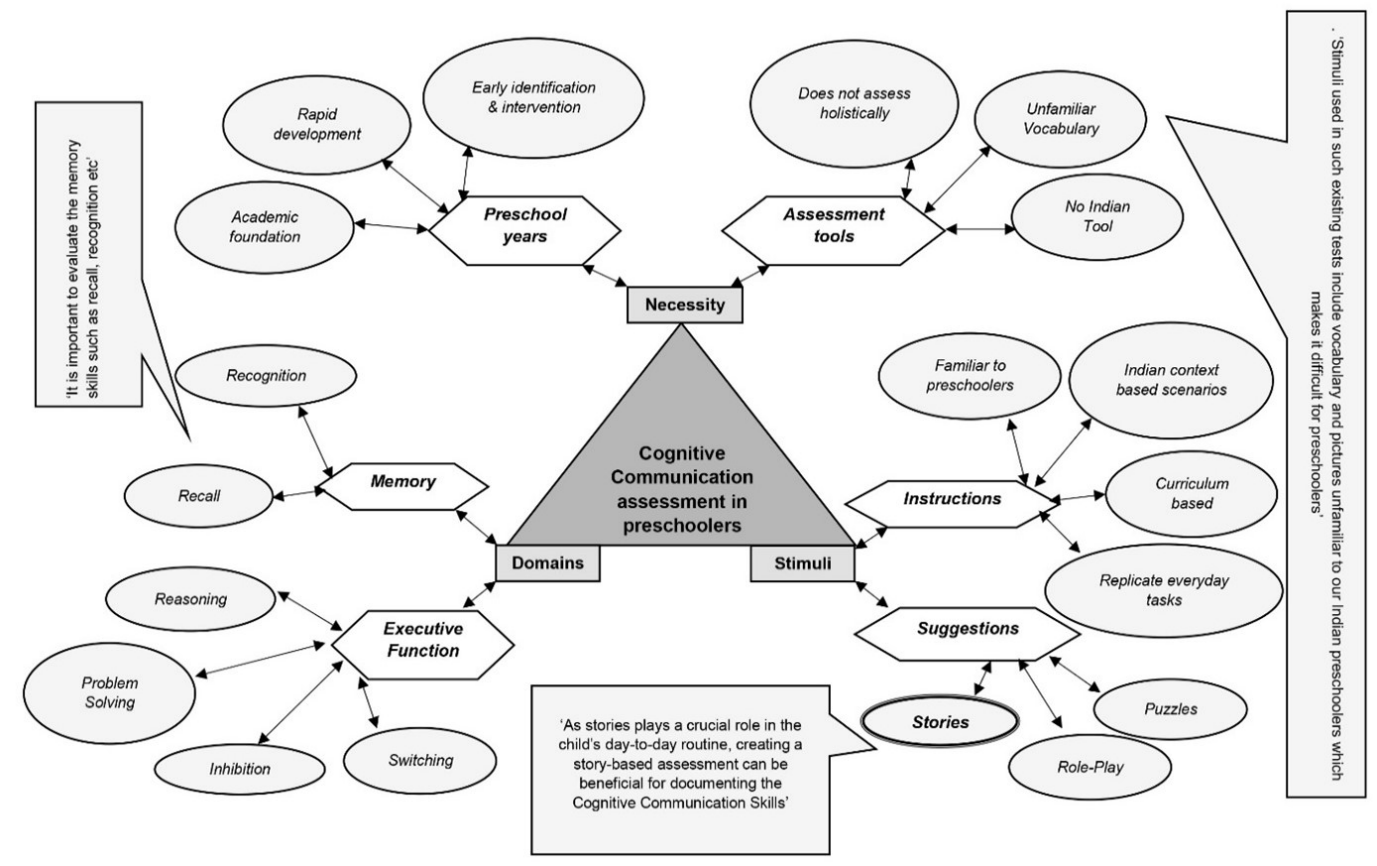
Themes, Codes, and Subcodes of Delphi Round One

The qualitative analysis of the responses during the first round of Delphi (as themes, codes, and sub codes), wherein ‘stories’ emerged as the most suitable stimuli along with ‘memory’ and ‘executive functions’ as important assessment domains among preschoolers, is illustrated in Figure 2.

**Figure 3:**
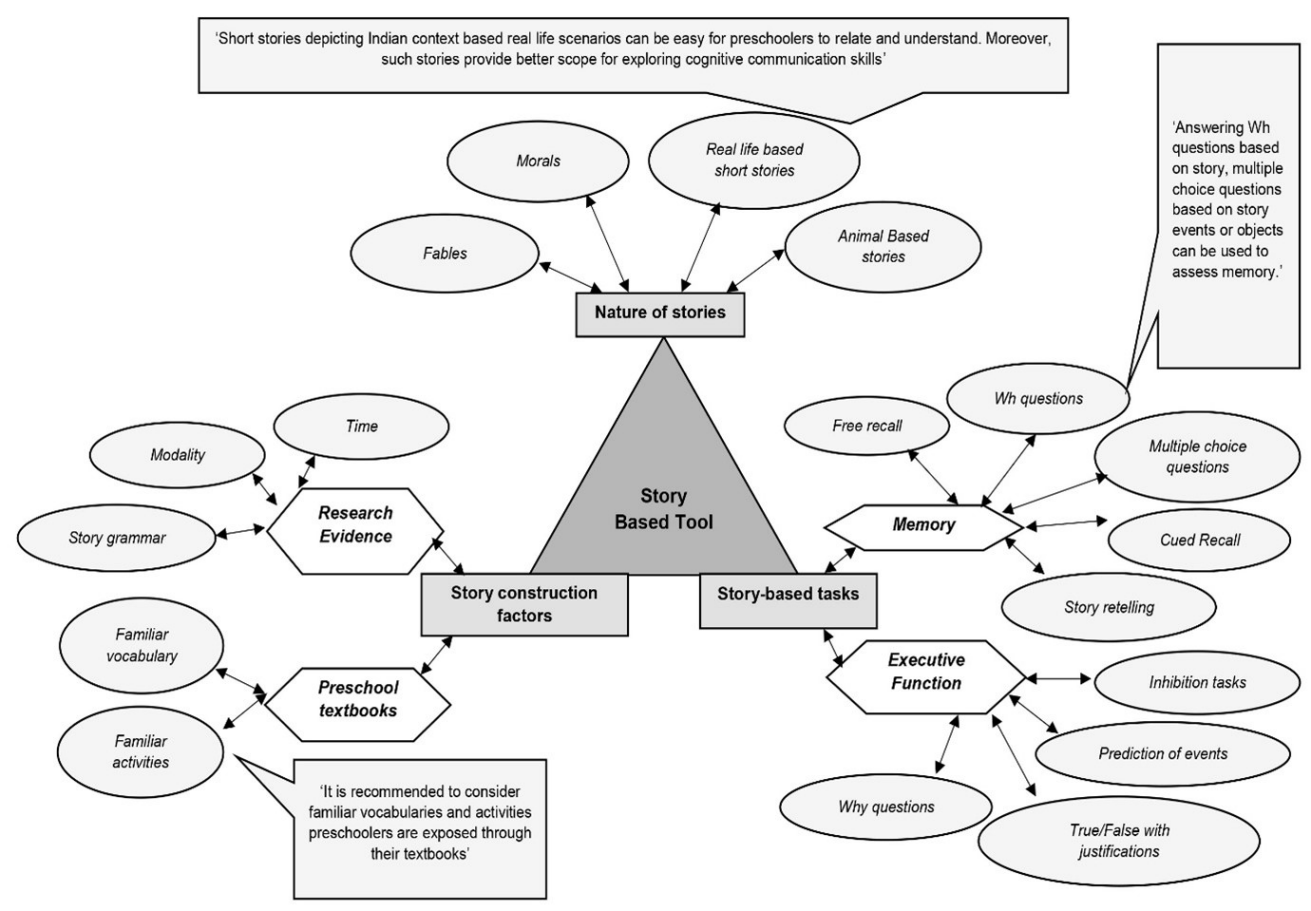
Themes, Codes and Subcodes of Delphi Round Two

During the second round of the Delphi, the experts emphasized the potential and intricacies of real-life short stories as suitable assessment stimuli for preschoolers and provided several recommendations regarding different cognitive tasks that could be considered around story-based stimuli. The qualitative analysis of the same, as themes, codes, and sub codes, is shown in Figure 3.

Based on the results obtained during first two rounds of Delphi and review of literature, the preliminary version of the tool was conceptualized around a story titled “A Day at Grandparents House” which had six picture-based illustrations and three memory (free recall, recognition, literary recall) and two executive function tasks (reasoning, inhibition, and switching) across three sections. This preliminary version of the story and the assessment tasks were content validated by six experts. Regarding the story, 100% consensus on agreement was obtained for all evaluation parameters except for the linguistic appropriateness of specific story contents and picture comprehensibility of one picture, which required modifications. Accordingly, three long sentences in the story were split into two each, as suggested by the experts, and a picture in the first story section was modified and redrawn to improve the clarity. Regarding the tasks, all the items under the free recall task reached greater than 80% agreement on every evaluation parameter and hence were maintained. In the recognition task, four items reached greater than 80% agreement. Two items did not reach 80% consensus, which required modification concerning the picture comprehensibility of the options, and were modified accordingly (For example, experts suggested to add grills to the picture of ‘window’, and spoon to the picture of ‘soup’ to improve comprehensibility among preschoolers). Four items belonging to the literary recall task did not reach an 80% consensus concerning linguistic appropriateness and picture comprehensibility (For example, the question ‘From which side of the house the sound was heard? with options, ‘Back, Front, Side’ was suggested to be modified as ‘From which part of the house the sound was heard? with options, ‘Back, Front, Side’ to reduce ambiguity and improve comprehensibility). The items were reformulated according to experts' suggestions, and the remaining eight items were maintained in their original format. In the reasoning task, two questions under explanation did not reach greater than 80% consensus, as there were duplicates, and hence were modified according to the replacement suggestions (For example, the explanation based question ‘Why did Virat run towards grandparents and hug them?’ was redundant with the inference based question, ‘Did Virat like grandparents? What made you feel so?’ and hence was suggested to be replaced by a different explanation based question targeting another story element). Three items in the inference task did not reach 20% agreement and were removed. The other 16 items under the reasoning task were maintained in the original format, as they obtained 100% agreement on all the evaluation parameters. All conditions under the inhibition and switching tasks obtained greater than 80% agreement on all evaluation parameters and were retained in the original format. The revised version of the story, along with the tasks, was again sent out for content validation and received greater than 80% consensus on agreement across all modified items. The content validity index of the revised version of the tool was greater than 0.78^(23)^ for all items.

The results of the pilot testing of the content-validated tool revealed that all participants could complete the tasks in approximately one hour. The total score obtained for each task was significantly higher in Group II than in Group I across all tasks (free recall [t(18)=-6.76,p<0.001], recognition [U=15, p<0.05], literary recall [U=0.5, p<0.001], reasoning [ t(18)=-8.06,p<0.001], and inhibition and switching [U=7.5, p<0.001]), confirming the appropriateness of the tasks to track the changes in cognitive-communication skills across the age groups. The descriptive results of the same have been summarized in Table 4. Hence, no modifications were required for the tool, and the final tool was named ‘Cognitive Communication Assessment in Preschoolers ‘ (CCAP).

**Table 4:** Results of Pilot Study

**Domain**	**Task**	**Group I (10) (M=3.8, SD=0.26)**		**Group II (10) (M=5.1, SD=0.19)**		**Age group wise comparison**
		**M(SD)**	**Median (IQR)**	**M(SD)**	**Median (IQR)**	**p-value (t-test or U-test)**
**Memory**	Free recall	7.10(6.24)	4(9)	50.50(19.28)	48(19.28)	t(18)=6.76,p<0.001
	Recognition	2.60(0.96)	2.50(1)	4.40(1.35)	4.5(3)	U=15, p<0.05
	Literary recall	4.80(1.39)	5(3)	9.30(1.63)	9(3)	U=0.5, p<0.001
**Executive Function**	Reasoning	8.10(1.19)	8(2)	16.30(2.98)	16.50(4)	t(18)=8.06,p<0.001
	Inhibition and switching	45(5.22)	46(6)	58.60(6.48)	60.50(8)	U=7.5, p<0.001

Caption: IQR = Interquartile range is the difference between third quartile (Q3) and first quartile (Q1) of the data

The final tool consisted of a story of 236-word length divided across three sections with corresponding six pictures and tasks assessing memory and executive functions. An overview of the finalized tool, along with examples of the tasks, is provided in Table 5.

**Table 5:** The Story and Task Details of the Final Tool CCAP with Examples

Story Details		Memory tasks			Executive Function tasks						
Theme of the Story	**Story elements**	**Free recall**	**Recognition**	**Literary recall**	**Reasoning**			**Inhibition and Switching**			
					**Explanation**	**Prediction**	**Inference**	**Naming**	**Inhibition**	**Switching**	**Inhibition & Switching**
'Boy visiting grandparents’ house gets scared by a dog. Later Boy happens to help the dog in the rain and dog saves the boy from a robber attack, and thus they become friends'	42 elements	3 questions	6 questions	12 questions	6 questions	6 questions	6 questions	4 practice and 16 test trials with picture presentations	4 practice and 16 test trials with picture presentations	4 practice and 16 test trials with picture presentations	4 practice and 16 test trials with picture presentations
	21 elements presented in Auditory modality and 21 elements presented in Auditory Visual modality	**Example:** What all do you remember from the story?	**Example:** Tell / Show me the object which was there in the story.(Cage, Bone, Jail, Soup)	**Example**: Who was waiting for Virat at the gate? (Parents, Grandparents, Uncle)	**Example:** Why did Virat get scared?	**Example:** What would have happened if grandpa didn’t lock the dog in the cage?	**Example**: Did Virat like grandparents? What made you feel so?	**Example:** Name picture by story character name	**Example:** Alternate between the story character names	**Example:** Name a story character by character name and other story character by target colour	**Example:** Alternate between name of the story character while substituting one of the character name with target colour

## DISCUSSION

The current study developed a tool, Cognitive Communication Assessment in Preschoolers (CCAP), to explore the cognitive-communication skills of preschoolers in the Indian context. The CCAP tool was developed, and content was validated using the Delphi method, which is considered the optimal method to obtain the most reliable consensus among a group of experts^(11)^ and, seeking consensus from experts on test contents has been recommended by Standard international procedures for test construction^(26)^ to obtain the content validity of developing tools. The use of the Delphi method in the current study aided in the structured development process and provided significant evidence for the content validity of the CCAP based on experts' views.

The CCAP incorporates the major domains of cognitive-communication skills, such as memory and executive function, which play a significant role in preschool children’s daily and academic lives. The literature shows that the verbal memory skills and academic skills of a child are closely related, and that the memory skills of a child predict literacy skills^(27)^. Likewise, executive function skills also play a vital role in children's reading and writing skills as young as preschool years^(4)^, and research supports that executive function skills, such as inhibitory control and working memory, are significantly related to emergent literacy skills and their future success in scholastic life^(5)^. Memory and executive function skills rapidly change during preschool years^(3,4)^, strongly indicating the need for assessment during this period. Experts in the first Delphi round of the current study also reported similar views, which led to the inclusion of memory and executive functions as the core assessment domains in the developed tool.

Stories have emerged as the most recommended and suitable stimuli to assess preschoolers' cognitive-communication skills by experts. Existing literature has also supported the use of story-based tasks to effectively document the development of cognitive-communication skills^(28)^. Stories that are common in preschool children's lives are an educational tool enriched with language and cognitive resources^(14)^. It is one of the most widely used academic stimuli, as story-based activities promote children’s thinking and literacy skills^(29)^. Stories help to bring children’s attention and interest to an activity; therefore, conducting assessments based on stories might ensure the completion of the assessment tasks in preschoolers, which was also evident in the pilot testing of the current story-based tool.

The story constructed for the developed tool incorporated the Indian context as suitable, appropriate, and relatable for Indian preschoolers. The story used in the present tool was constructed based on natural life sequences, considering the reality-prone nature of preschoolers^(15)^. The pictures, objects, characters, dresses, sequences, and task formats used in the story were considered to reflect the Indian context, which is familiar to preschoolers. The review of preschool textbooks facilitated the selection of familiar vocabulary for Indian preschoolers, and the story structure followed story grammar, logical structure, and psychological causality to facilitate better understanding and recall^(16)^. The story also incorporated an animal character in the storyline, as most storybooks for preschool children used animal characters to attract children's attention and interest^(30)^. Although story-based tasks have been used in previous studies to explore cognitive skills in preschoolers, they were restricted to a specific cognitive domain^(25,28)^. In contrast with such tasks, the current tool provides an opportunity to explore the cognitive-communication skills widely in preschoolers for the memory and executive function domains using real-world living context-based story tasks.

Although the method of developing the tool makes it ideal for assessing cognitive-communication skills in preschoolers in the Indian context by following the Indian contextual, cultural, and educational framework, the tool has certain limitation. Though every attempt was made to ensure diversity within the expert panel, individual panellist’s biases and the expert panel's size might have influenced the content evaluation of the items included in the tool.

## CONCLUSION

The present research describes a Delphi-based development and validation of a ‘Cognitive-Communication Assessment Tool’ for Indian preschoolers. The study highlights the utility of a Delphi survey-based method in tool development, which offers a systematic approach from conceptualization to validation of the tool by building consensus among different stakeholders. Although the protocol is specially designed for the Indian context, the detailed description of the methods could be helpful for other researchers in the field who wish to develop instruments like this. Further investigations regarding the efficacy of the developed tool to profile cognitive-communication skills in Indian preschoolers and its psychometric properties are in progress. The tool, though presently designed specifically for preschoolers of Indian origin, could be explored by researchers elsewhere for translation and cross-cultural adaptation with adequate validation and analysis of psychometric properties.
